# A Case of Abnormal Origin of the Azygos Vein System: An Anatomical Study in a Human Cadaver

**DOI:** 10.7759/cureus.5068

**Published:** 2019-07-02

**Authors:** Konstantinos N Koutsouflianiotis, George K Paraskevas, Panagiotis Kitsoulis, George Noussios

**Affiliations:** 1 Internal Medicine, General Hospital of Thessaloniki "G. Gennimatas", Thessaloniki, GRC; 2 Orthopaedics, Aristotle University of Thessaloniki, Thessaloniki, GRC; 3 Orthopaedics, University of Ioannina, Ioannina, GRC; 4 Physical Education and Sports Sciences, Aristotle University of Thessaloniki, Thessaloniki, GRC

**Keywords:** azygos vein, hemiazygos vein, anatomical variation, anatomical dissection, abnormal origin, azygos vein system

## Abstract

The azygos vein (AV) system is considered a venous system which displays great variability in its formation and course. Especially, regarding the origin of the AV system, the international literature describes mostly the union of the ascending lumbar with the subcostal veins, though other origins are documented as well. The current study displays an abnormal origin of the azygos system, which to the best of our knowledge has never been described before.

## Introduction

The origin of both azygos vein (AV) and hemiazygos vein (HeAV) is in the union of the ascending lumbar and the subcostal veins. The AV ascends in the posterior mediastinum after it passes through the diaphragm, whilst both HeAV and accessory HeAV lie on the left side of the vertebral column in the mediastinum [1-2]. However, on searching the relative international anatomical literature, one will reach the conclusion that the origin of the AV system is not constant, but displays a great variability. Certain patterns are described with lateral, intermediate and/or medial roots, which include ascending lumbar vein, inferior vena cava, first lumbar vein or renal vein as the origin of the AV system [3]. 

In the current study, we present a case of an abnormal origin of the azygos venous system which cannot be found, to the best of our knowledge, in the reviewed literature. 

## Case presentation

**Figure 1:**
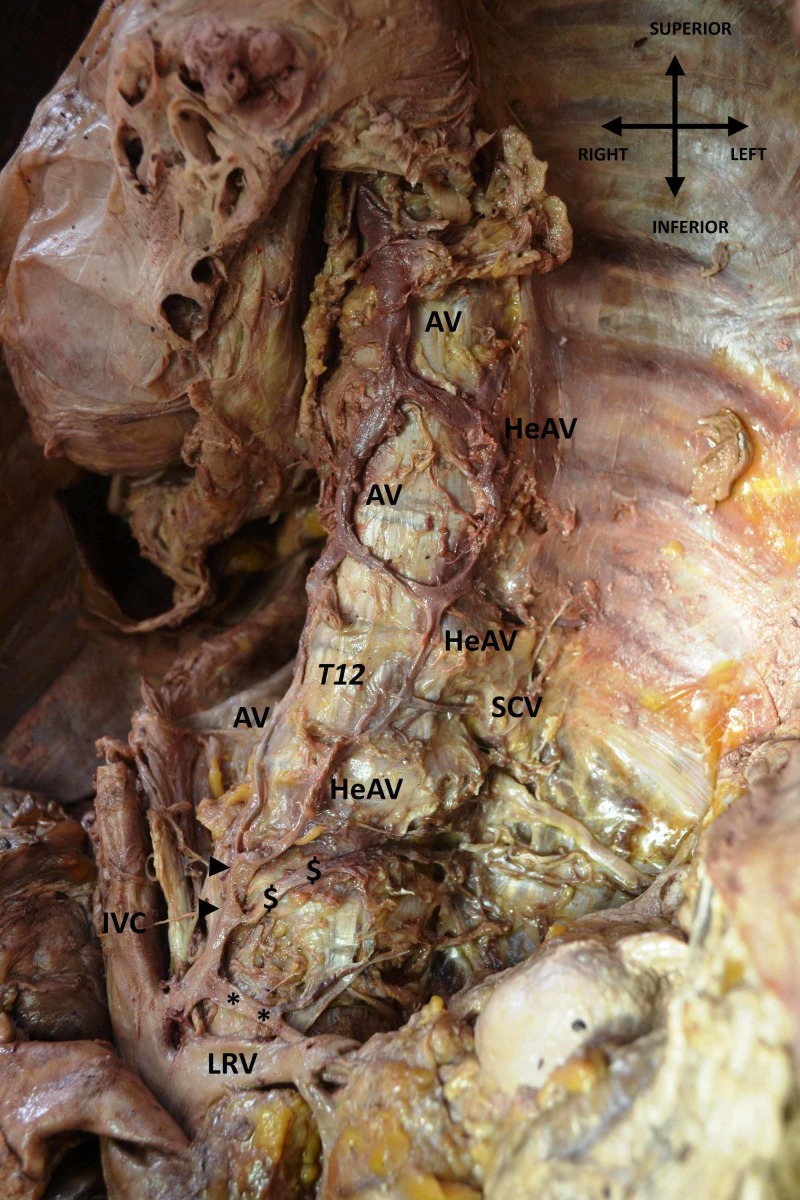
Abnormal origin of azygos vein (AV) system The AV and hemiazygos vein (HeAV) are seen to be originating from the same venous branch (arrowheads), which comes from the inferior vena cava (IVC). The figure depicts HeAV being formed by the union with the left subcostal vein (SCV) on the twelfth thoracic vertebra (T12). LRV: left renal vein; *: left adrenal vein; $: left first lumbar vein.

During a routine dissection in our Department of Anatomy and Surgical Anatomy, we encountered a very rare case of abnormal origin of the AV system (Figure 1).

The dissection was conducted on a 68-year-old formalin-fixed male cadaver, used for educational and research purposes, whose death was unrelated to the present case report. Specifically, after meticulous dissection of the thorax region and the mediastinum, and after the excision of both lungs and the heart by means of the classical method of anatomical dissection, we detected both AV and HeAV originating from the same venous branch, which originated from the inferior vena cava (IVC). To the formation of the AV and HeAV participated the subcostal veins, the right and the left, respectively. The diameter of the AV on the twelfth thoracic vertebra was 3.59 mm, while the diameter of the HeAV on the same vertebra was 2.14 mm. Our findings were documented by several photographs taken using a Nikon D3100 digital camera, and the measurements were made using a digital vernier caliper with an accuracy of 0.01 mm. No other congenital anomalies, variations or pathological conditions, or evidence of previous surgical interventions in the region were present.

## Discussion

The origin of the azygos venous system is not constant. Both AV and HeAV are often formed by the union of the ascending lumbar veins with the subcostal veins. AV penetrates the diaphragm mostly through the aortic opening and it ascends in the posterior mediastinum, passing on the right side of the inferior eight thoracic vertebrae, arching over the root of the right lung to join the superior vena cava [1]. On the other side, the HeAV inferiorly and the accessory hemiazygos vein superiorly, lie longitudinally on the left side of the thoracic vertebrae [2].

From the embryological aspect, AV is formed by the union of the right supracardinal vein with a portion of the posterior cardinal vein, while on the left side, the left supracardinal vein forms the HeAV which empties into the AV. The intercostals veins on both sides enter into the right and left supracardinal veins respectively [4].

To the best of our knowledge, few published studies can be found regarding the origin of the azygos venous system. In specific, Alves et al., after dissecting 30 cadavers came across 13 cases (43.33%) of AV originating from the right subcostal vein, three cases (10.00%) from right subcostal and ascending lumbar vein, three cases (10.00%) from right subcostal vein with contribution from IVC and three cases (10.00%) of AV originating from right subcostal vein with contribution from IVC and ascending lumbar vein. The rest of the cadavers were found to have an origin of AV formed by a combination of the above veins and completely different from our case, thus an origin of both AV and HeAV from the same branch which came from IVC [5]. On the other hand, Seib after dissecting 190 cadavers found in 85% of the cases (162 cadavers) both AV and HeAV originating from the union of the ascending lumbar vein with the subcostal vein bilaterally. Seib also found AV and HeAV arising from one, two, or three roots (lateral, intermediate, and medial), although the lateral root seemed to be more constant [6].

In classical anatomical textbooks, the origin of AV and HeAV is reported mostly by the union of the subcostal with the ascending lumbar veins [1-3,7-9], while in Moore’s Anatomy AV came from the IVC and HeAV from the left ascending lumbar vein [10]. As we searched through the literature, our case could be considered as a unique one, since the azygos venous system came from the same venous branch, originating from IVC. It is a case that can be added as a variation to a venous system which displays high variability, a knowledge necessary to the modern anatomist and surgeon of the region.

## Conclusions

Anatomical variations are considered a matter of high importance since the knowledge of abnormalities can assist the surgeon of each region to be more accurate during surgical interventions. The origin of the AV system needs further clarification since there are cases of abnormal origin that still remain undocumented.
